# Visible and infrared emission from Si/Ge nanowires synthesized by metal-assisted wet etching

**DOI:** 10.1186/1556-276X-9-74

**Published:** 2014-02-12

**Authors:** Alessia Irrera, Pietro Artoni, Valeria Fioravanti, Giorgia Franzò, Barbara Fazio, Paolo Musumeci, Simona Boninelli, Giuliana Impellizzeri, Antonio Terrasi, Francesco Priolo, Fabio Iacona

**Affiliations:** 1 IPCF CNR, viale F. Stagno d'Alcontres 37, Faro Superiore, Messina 98158, Italy; 2 MATIS IMM CNR, via Santa Sofia 64, Catania 95123, Italy; 3 Dipartimento di Fisica e Astronomia, Università di Catania, via Santa Sofia 64, Catania 95123, Italy; 4 Scuola Superiore di Catania, Università di Catania, via Valdisavoia 9, Catania 95123, Italy

**Keywords:** Nanowires, Photoluminescence, Semiconductor nanostructures

## Abstract

**Abstract:**

Multi-quantum well Si/Ge nanowires (NWs) were realized by combining molecular beam epitaxy deposition and metal-assisted wet etching, which is a low-cost technique for the synthesis of extremely dense (about 10^11^ cm^−2^) arrays of NWs with a high and controllable aspect ratio. In particular, we prepared ultrathin Si/Ge NWs having a mean diameter of about 8 nm and lengths spanning from 1.0 to 2.7 μm. NW diameter is compatible with the occurrence of quantum confinement effects and, accordingly, we observed light emission assignable to the presence of Si and Ge nanostructures. We performed a detailed study of the photoluminescence properties of the NWs, with particular attention to the excitation and de-excitation properties as a function of the temperature and of the excitation photon flux, evaluating the excitation cross section and investigating the presence of non-radiative phenomena.

**PACS:**

61.46.Km; 78.55.-m; 78.67.Lt

## Background

Semiconductor nanowires (NWs) represent a very promising material to become the building blocks for future electronic [1] and photonic [2,3] devices, photovoltaic cells [3,4], and sensors [5]. Further unexpected applications can be foreseen by fully exploiting the enhanced potentialities of NWs composed by more than a single semiconductor; within this context, the presence of Si/Ge multi-quantum wells (MQWs) inside a NW could be particularly intriguing because it allows putting together two different confined semiconductors, which absorb and emit photons at different wavelengths. In spite their enormous potentialities, the current research on Si/Ge NWs is still in a quite preliminary stage, mainly as far as their light emission properties are concerned [6], due to the difficulties involved with their synthesis. In fact, ‘bottom-up’ approaches based on the vapor–liquid-solid growth (VLS) mechanism [7], due to the presence of the Gibbs-Thomson effect, do not allow obtaining the NW diameter values (lower than 10 nm) which are necessary to observe light emission. Furthermore, the metal catalyst (generally Au) used in VLS-based approaches is usually incorporated inside the growing NWs, acting as a deep non-radiative recombination center, negatively altering both electrical and optical properties [8].

Metal-assisted wet etching processes were recently proposed as a very promising alternative method for the synthesis of Si NWs having a size compatible with the occurrence of quantum confinement effects [9,10]. In these processes, the role of metal is to catalyze Si oxidation induced by H_2_O_2_; afterwards, SiO_2_, selectively formed where metal and Si are in contact, is etched by HF. Metal catalysts are usually added to the etching solution as a salt (typically AgNO_3_) [10]; however, this approach leads to the formation of dendrites, whose subsequent removal can damage the NWs [10]. Note also that NWs with sizes compatible with quantum confinement effects were never obtained by etching processes assisted by metal salts [11]. Recently, we proposed a modified metal-assisted wet etching process, in which the salt was replaced by a thin metal film [2,12,13]. This process was demonstrated to be a fast and low-cost technique to fabricate Si NWs since it does not require any kind of expensive and time-consuming lithographic techniques. It also allows the control of several structural parameters like aspect ratio, diameter, density, orientation, and doping type and level; in particular, a unique feature of this process is the possibility to obtain NWs with an extremely small diameter, such as to exhibit a strong light emission at room temperature due to quantum confinement effects [2,12]. Moreover, since metal-assisted etching is accomplished at room temperature, metal is not incorporated inside the NWs, but it remains trapped at the bottom of the etched regions and can be easily removed by an appropriate etching solution. Metal-assisted wet etching was already used for the synthesis of Si/Ge NWs; the proposed procedure involves the pre-patterning of the substrate by anodic aluminium oxide masks, but no light emission was reported [14].

In this paper, we demonstrate that it is possible to synthesize light-emitting Si/Ge NWs by metal-assisted wet etching of Si/Ge MQW grown by molecular beam epitaxy (MBE) on a Si substrate. We report a detailed study on the structural and optical properties of this system which, remarkably, exhibits both visible (due to Si) and infrared (IR; due to Ge) light emissions.

## Methods

**Figure 1:**
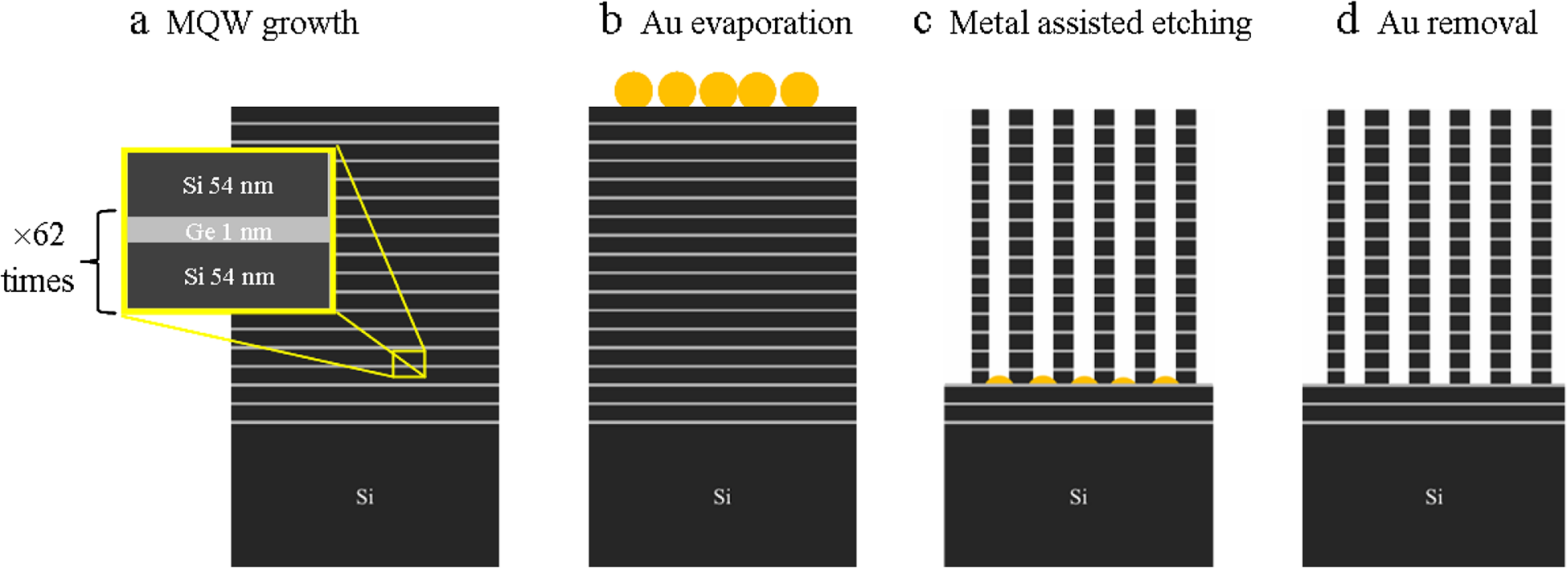
**Scheme of the fabrication of Si/Ge NWs. (a)** The starting MQW consists of alternating 1-nm-thick Ge layers and 54-nm-thick Si layers, grown by MBE. This unit is repeated 62 times. **(b)** Deposition of an Au thin layer (2 nm) by EBE. **(c)** Formation of Si/Ge NWs by dipping the sample in an aqueous solution of HF and H_2_O_2_. **(d)** Removal of Au particles by using an aqueous solution of KI + I_2_. Steps (b,c,d) are performed at room temperature.

**Figure 2:**
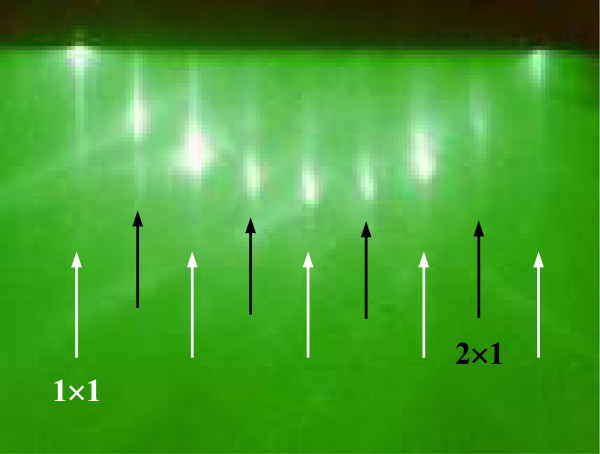
**RHEED analysis of the grown MQW.** The image shows spots of the 2 × 1 surface reconstruction (black arrows) superimposed to those of the initial 1 × 1 symmetry (white arrows). The presence of this diffraction pattern guarantees for a clean surface and a good two-dimensional epitaxial growth.

Si/Ge NWs were obtained starting from a Si/Ge MQW grown by MBE on a (001) Si substrate at a temperature of 450°C, consisting of alternating Si (54-nm thick) and Ge (1-nm thick) layers (Figure 1a) deposited at a rate of 0.3 and 0.01 nm · s^−1^, respectively. The Si/Ge stack is repeated 62 times, giving an overall sample thickness of about 3.5 μm. Due to the relatively low-growth temperature, the Ge layers show an excellent pseudomorphic two-dimensional heteroepitaxy, as demonstrated by the *in situ* reflection high-energy electron diffraction (RHEED) image shown in Figure 2, while a transition to Stransky-Krastanov Ge island regime would have been taken place for the same Ge thickness at higher temperatures [15]. The samples were UV oxidized and dipped in 5% HF to obtain a clean and oxide-free surface. Afterward, a thin Au layer, having a thickness of 2 nm, was deposited on the MQWs at room temperature by electron beam evaporation (EBE), by using high-purity (99.9%) Au pellets as a source (Figure 1b). After Au deposition, the sample surface consisted of nanometric uncovered Si areas, almost circular and totally embedded within the Au regions. The samples were then etched at room temperature at a rate of 0.13 μm · min^−1^ in an aqueous solution of HF (5 M) and H_2_O_2_ (0.44 M) to form Si/Ge NWs (Figure 1c). Finally, the removal of the Au particles was carried out by dipping the sample in a KI + I_2_ aqueous solution (Figure 1d).

NW structural characterization was performed by scanning electron microscopy (SEM) and Raman spectroscopy. SEM analyses were performed using a field emission Zeiss Supra 25 microscope (Oberkochen, Germany). Micro-Raman spectra were acquired using a HR800 spectrometer (Horiba-Jobin Yvon, Kyoto, Japan), exciting the system with the 364-nm line of an Ar^+^ laser, that allows to avoid the spectral contribution of the substrate due to its low penetration depth in Si (about 12 nm). The exciting beam has a power of 20 μW to prevent heating effects and it was focused on the sample with about 1 μm^2^ spot area through a fluorinated × 60 (NA = 0.9) Olympus microscope objective (Tokyo, Japan).

Photoluminescence (PL) measurements were performed by pumping with the 488-nm line of an Ar^+^ laser. Pump power was varied from 1 to 200 mW, corresponding to a photon flux *φ* ranging from 3.1 × 10^19^ to 6.2 × 10^21^ cm^−2^ · s^−1^, and the laser beam was chopped through an acousto-optic modulator at a frequency of 55 Hz. The PL signal was analyzed by a single-grating monochromator and detected by a photomultiplier tube in the visible and by a liquid-nitrogen-cooled Ge detector or an IR-extended photomultiplier tube in the IR. Spectra were recorded with a lock-in amplifier using the acousto-optic modulator frequency as a reference. Time-resolved measurements were made by pumping the system at a steady state, then switching off the laser beam, and detecting how the PL signal at a fixed wavelength decreases as a function of time. The overall time resolution of the system is 200 ns. Low-temperature measurements were performed by using a closed cycle He cryostat with the samples kept in vacuum at a pressure of 10^−5^ Torr.

## Results and discussion

**Figure 3:**
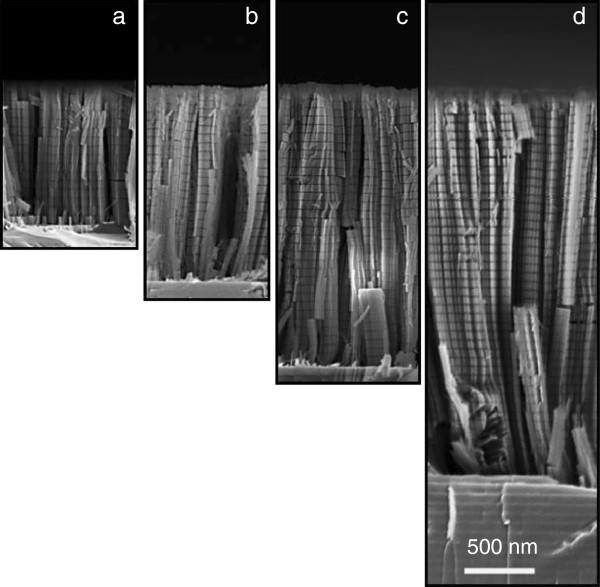
**Cross-sectional SEM analysis of MQW Si/Ge NWs.** The images show NWs having lengths **(a)** 1.0, **(b)** 1.7, **(c)** 2.0, and **(d)** 2.7 μm.

Figure 3a,b,c,d reports cross-sectional SEM images of Si/Ge NWs with different lengths obtained by the above-described metal-assisted wet etching approach by using increasing etching times. The images display dense (about 10^11^ NWs · cm^−2^ can be counted in plain view; SEM images here not shown) and uniform arrays of NWs; the length ranges from 1.0 (Figure 3a) to 2.7 μm (Figure 3d) and linearly depends on the etching time.

**Figure 4:**
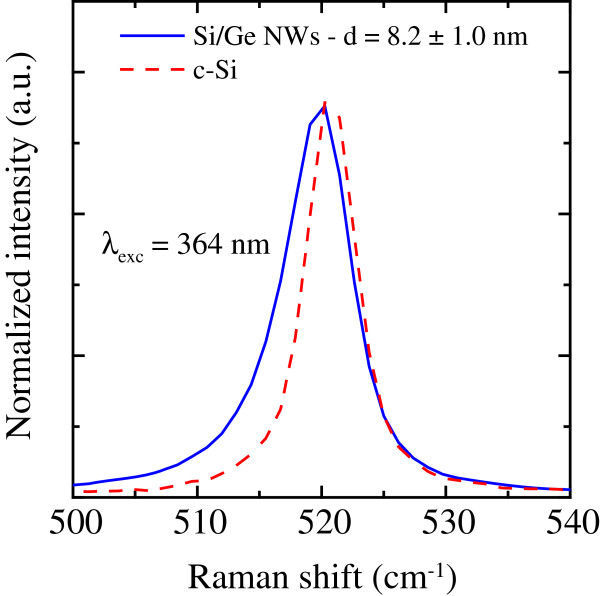
**Raman analysis of Si/Ge NWs.** Comparison between the Raman spectra of Si/Ge NWs (blue continuous line) and bulk crystalline Si (red dashed line). A fit to the spectrum of Si/Ge NWs gives a diameter mean value of 8.2 ± 1.0 nm.

Raman measurements were used to estimate the NW mean size. Figure 4 shows the typical asymmetrically broadened Raman peak (solid line), due to the Si-Si stretching mode in optically confined crystalline Si nanostructures, detected on the Si/Ge NWs. The peak appears red shifted with respect to the symmetric and sharper peak typical of bulk crystalline Si at 520 cm^−1^ (dashed line), reported in the same figure for comparison. The peak was fitted using a phenomenological model developed by Richter [16] and Campbell and Fauchet [17] for strongly confined phonons in nanocrystals and more recently adapted to Si NWs [2,18]. The fit procedure gives a NW diameter of 8.2 ± 1.0 nm.

**Figure 5:**
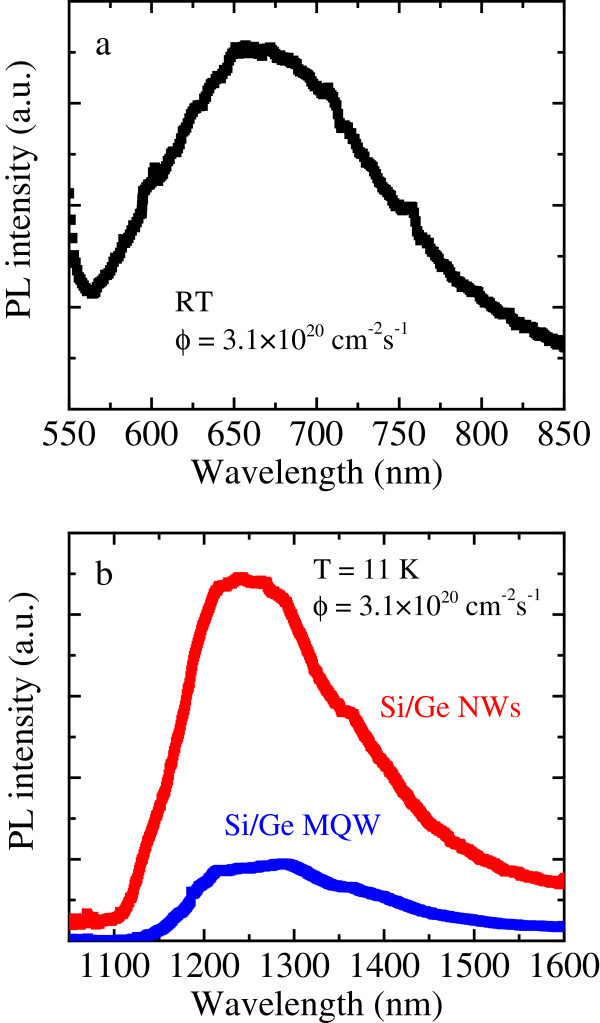
**PL spectra of Si/Ge NWs. (a)** Room temperature spectrum in the visible region. **(b)** Spectrum in the IR region obtained at 11 K. Both spectra were obtained with a photon flux of 3.1 × 10^20^ cm^−2^ · s^−1^.

We studied the PL properties of the Si/Ge NWs having a length of 2.7 μm. In agreement with the extremely small diameter obtained, an intense room temperature PL coming from quantum-confined Si nanostructures occurs under a 488-nm excitation, as shown in Figure 5a; the PL spectrum consists of a broadband centered at about 670 nm which strongly resembles that one previously observed and reported for pure Si NWs [2,12]. A similar PL spectrum, although less intense, was observed in shorter NWs. No Ge-related PL signals are detected in the IR region at room temperature.

Relevant variations of the PL spectrum are found by decreasing the temperature down to 11 K. Indeed, the intensity of the Si-related signal strongly decreases by decreasing temperature, as previously reported in the case of pure Si NWs [12]. On the other hand, a PL signal appears in the IR region at about 1,240 nm (red squares), as shown in Figure 5b. The peak position is in agreement with literature data concerning light emission from Ge nanostructures [19-21]. It is noteworthy that the emission is enhanced by about a factor of 5 with respect to that one coming from the unetched MQW, shown in the same figure as blue squares, which suggests that stronger quantum confinement effects are operating in the NWs (where Ge regions can be considered as nanodots) with respect to the MQW. To this end, we also underline that NWs cover only about the 50% of the sample surface, so that the actual enhancement factor of the PL intensity for Si/Ge NWs accounts for at least an order of magnitude. Although ultrathin Si/Ge NWs were already successfully synthesized [6,14], to our knowledge, the above-reported data constitute the first evidence of simultaneous light emission from both Si and Ge nanostructures in Si/Ge NWs.

**Figure 6:**
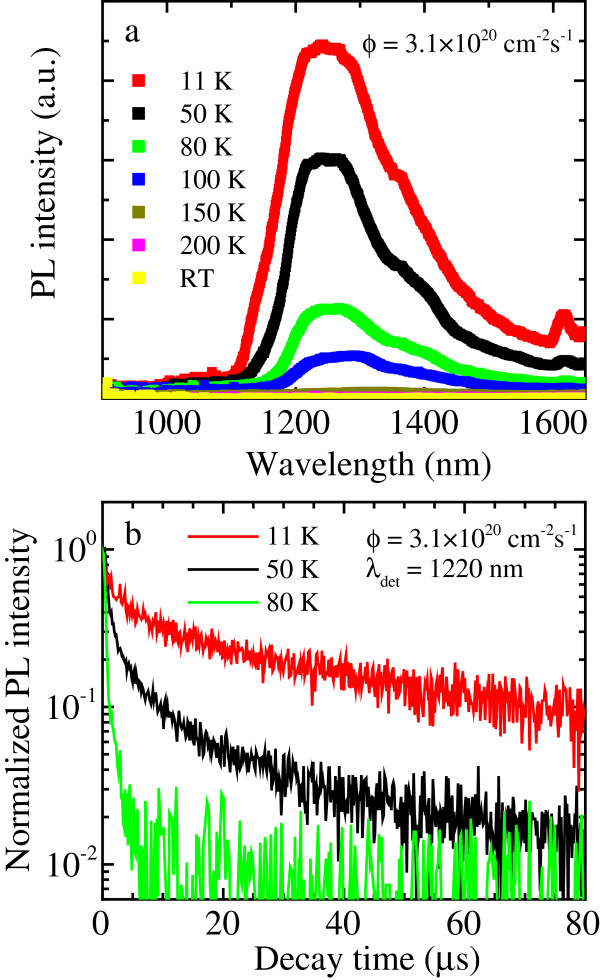
**PL properties of Si/Ge NWs as a function of temperature. (a)** PL spectra in the IR region of Si/Ge NWs from 11 K to room temperature. **(b)** PL time-decay curves measured at 1,220 nm and at temperatures in the 11- to 80-K range. All measurements were performed with a photon flux of 3.1 × 10^20^ cm^−2^ · s^−1^.

Since the properties of the Si-related PL signal observed in Si/Ge NWs tightly resemble those found in pure Si NWs [2,12], in the rest of the work, we mainly focused our attention on the Ge-related emission. In particular, we studied in detail the IR PL emission as a function of the temperature, as reported in Figure 6a. We observed that by decreasing the temperature, the PL intensity monotonically increases, due to a reduced efficiency of non-radiative phenomena. Furthermore, it can be noticed that the PL emission exhibits a blueshift toward shorter wavelengths by decreasing temperature, in agreement with the well-known dependence of the Ge bandgap on temperature.

We also measured the time-decay curves of the Ge-related PL signal at 1,220 nm as a function of the temperature, as shown in Figure 6b. Decays become faster by increasing the temperature and cannot be fitted by a single exponential function, so that lifetime (*τ*) values were evaluated by taking the time at which the PL signal becomes 1/e of its initial value. The observed decreasing *τ* values from 7.0 μs at 11 K to 0.6 μs at 80 K provide a clear evidence that non-radiative phenomena occur and quench the luminescence. This behaviour is a clear indication of the fact that fast non-radiative phenomena, such as Auger processes or thermally activated quenching processes [22], influence the de-excitation of Si/Ge NWs. The efficiency of such processes increases by increasing the temperature; indeed, they are able to completely quench the IR PL signal at room temperature.

**Figure 7:**
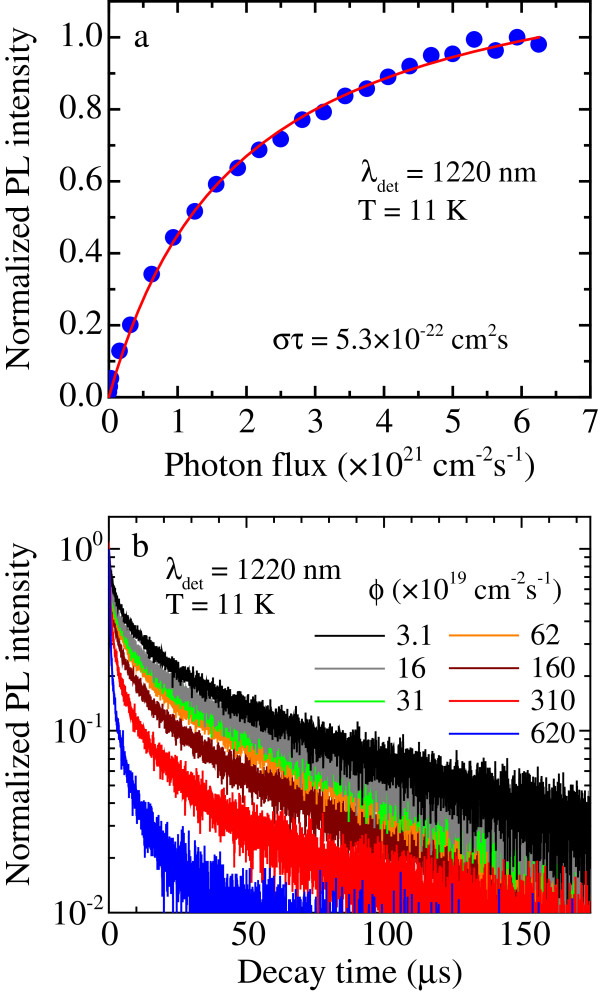
**PL properties of Si/Ge NWs as a function of photon flux. (a)** PL intensity at 1,220 nm detected at 11 K as a function of the photon flux. The red line is a fit to the data. **(b)** Time-decay curves of the PL signal at 1,220 nm performed at 11 K and for different photon fluxes.

We also analyzed the dependence of the Ge-related PL signal, detected at 11 K, on the photon flux. As shown in Figure 7a, the PL intensity at 1,220 nm increases by increasing the excitation photon flux from 3.1 × 10^19^ to 6.2 × 10^21^ cm^−2^ · s^−1^, due to the increase of the number of e-h pairs generated into the wires; in addition, the figure evidences a sublinear behavior of the PL intensity as a function of the photon flux, which indeed clearly tends to a saturation value. We also analyzed the behaviour of the PL time-decay curves at 11 K as a function of the photon flux, as reported in Figure 7b. By increasing the photon flux, the lifetime decreases (*τ* is reduced from 8.7 to 0.5 μs) due to the occurrence of non-radiative phenomena and, in particular, of the Auger process.

The dependence of the PL intensity on the excitation photon flux can be understood by solving the rate equation that describes the excitation and de-excitation processes of excitons in the Si/Ge NWs:

(1) $$\frac{dN^{*}}{\mathrm{dt}}=\sigma \phi \left(N-N^{*}\right)-\frac{N^{*}}{\tau }$$

where *N* is the total amount of excitable emitting centers, N* is the excited emitting center population, *σ* is the excitation cross section, *φ* is the photon flux impinging on the sample, and *τ* is the lifetime of the excited state, taking into account both radiative and non-radiative processes.

At steady state, by solving Equation 1 and taking into account that the PL intensity (*I*_PL_) is proportional to N^*^/*τ*_rad_, where *τ*_rad_ is the radiative lifetime of the emitting center, we obtain

(2) $$\frac{I_{\mathrm{PL}}}{I_{{\mathrm{PL}}_{\text{max}}}}=\frac{\sigma \tau \varphi }{\sigma \tau \varphi +1},$$

where $I_{{\mathrm{PL}}_{\text{max}}}$ is the saturation value of *I*_PL_. From a fit to the data of Figure 7a by using Equation 2 (shown as a red line), we obtain a *στ* value of 5.3 × 10^−22^ cm^2^ · s^−1^. Since the lifetime value is 0.5 μs, the measured excitation cross section results to be 1.1 × 10^−15^ cm^2^. We remark that, in the presence of Auger processes, the measured lifetime is a function of the excitation density, so that the use of a constant lifetime in Equation 1 represents an approximation. Accordingly, the analysis shown in Figure 7a essentially leads to an estimate of the order of magnitude of the excitation cross section, which however results in good agreement with literature data on Ge nanostructures [23].

## Conclusions

We have demonstrated that a metal-assisted wet etching process can be effectively used to etch Si/Ge MQW and to produce ultrathin Si/Ge NWs which exhibit room temperature PL in the visible range, due to quantum-confined Si nanostructures, and low-temperature PL in the IR range, due to the nanometric Ge layers.

The IR PL emission from the Ge nanostructures is strongly influenced by the occurrence of non-radiative Auger processes, which determines a strong temperature quenching of the PL. In spite of this limitation, the capability of the metal-assisted wet etching technique to synthesize wires in which two semiconductors, characterized by different absorption and emission spectra, are put together opens the ways to new and unexpected applications of NWs in photonics and photovoltaics.

## Competing interests

The authors declare that they have no competing interests.

## Authors’ contributions

AI conceived the study, supervised all the experiments and participated in the writing of the paper. PA and VF synthesized the NWs, carried out the PL measurements and SEM characterization, and participated in data interpretation. GF carried out the PL measurements and participated in data interpretation. BF carried out and interpreted the Raman measurements. PM participated in NW synthesis and characterization. SB carried out the structural characterization of NWs. GI and AT realized and characterized the Si/Ge MQW. FP supervised the whole project. FI participated in data interpretation and wrote the paper. All authors read and approved the final manuscript.
